# Differential Expression of RAD51AP1 in Ovarian Cancer: Effects of siRNA In Vitro

**DOI:** 10.3390/jpm12020201

**Published:** 2022-02-01

**Authors:** Alice Filipe, Periklis Katopodis, Dimple Chudasama, Rachel Kerslake, Jeyarooban Jeyaneethi, Vladimir Anikin, Elisabete Silva, Ioannis Kyrou, Harpal S. Randeva, Cristina Sisu, Marcia Hall, Emmanouil Karteris

**Affiliations:** 1 Department of Life Sciences, Division of Biosciences, College of Health, Medicine and Life Sciences, Brunel University London, Uxbridge UB8 3PH, UK; alice.filipe@brunel.ac.uk (A.F.); periklis.katopodis@brunel.ac.uk (P.K.); dimpz22@hotmail.com (D.C.); Rachel.kerslake@brunel.ac.uk (R.K.); jeyarooban.jeyaneethi@brunel.ac.uk (J.J.); Elisabete.Silva@brunel.ac.uk (E.S.); cristina.sisu@brunel.ac.uk (C.S.); 2 Division of Thoracic Surgery, The Royal Brompton & Harefield NHS Foundation Trust, Harefield Hospital, Harefield UB9 6JH, UK; v.anikin@rbht.nhs.uk; 3 Department of Oncology and Reconstructive Surgery, Sechenov First Moscow State, Medical University, 119146 Moscow, Russia; 4 Warwickshire Institute for the Study of Diabetes, Endocrinology and Metabolism (WISDEM), University Hospitals Coventry and Warwickshire NHS Trust, Coventry CV2 2DX, UK; ioannis.Kyrou@uhcw.nhs.uk (I.K.); harpal.randeva@uhcw.nhs.uk (H.S.R.); 5 Warwick Medical School, University of Warwick, Coventry CV4 7AL, UK; 6 Centre for Sport, Exercise and Life Sciences, Research Institute for Health & Wellbeing, Coventry University, Coventry CV1 5FB, UK; 7 Aston Medical Research Institute, Aston Medical School, College of Health and Life Sciences, Aston University, Birmingham B4 7ET, UK; 8 Department of Food Science & Human Nutrition, School of Food and Nutritional Sciences, Agricultural University of Athens, 11855 Athens, Greece; 9 Mount Vernon Cancer Centre, Northwood, London HA6 2RN, UK

**Keywords:** ovarian cancer, RAD51AP1, biomarker, T2DM

## Abstract

Background: DNA double strand breaks can affect genome integrity potentially leading to cancer. RAD51-associated protein 1 (RAD51AP1), an accessory protein to RAD51, is critical for homologous recombination, a key DNA damage response pathway. Emerging studies indicate a novel role for RAD51AP1 in carcinogenesis. Here we provide additional insight into the role of RAD51AP1 in ovarian cancer (OvCa). Methods: Gene expression and patient phenotype data were obtained from TCGA and GTEX project consortia for bioinformatics analysis. Immunohistochemistry of OvCa tissue microarray was undertaken. Functional analyses were performed in a SKOV3 OvCa cell line with down-regulation of RAD51AP1 using siRNA. Results: RAD51AP1 is overexpressed at gene level in primary and recurrent OvCa compared to controls. At protein level, RAD51AP1 was up-regulated in low grade serous tumors compared to high grade OvCa. There was higher expression of RAD51AP1 in OvCa metastatic to lymph nodes compared to primary cancer samples. Gene enrichment analyses identified 12 differentially expressed genes (DEGs) related to OvCa, eight of which are also common in tissue from patients with type 2 diabetes mellitus (T2DM). Conclusions: RAD51AP1 is overexpressed in OvCa, Given the link between OvCa and T2DM, the eight-gene signature shows potential for predictive value.

## 1. Introduction

Double strand breaks (DSBs) are considered one of the most dangerous types of DNA damage, with the subsequent loss of genome integrity potentially leading to cancer. Cells have a functional capacity to respond to such deleterious insults via homologous recombination (HR) DNA repair. This highly conserved process is critical for reducing the risk of carcinogenesis [1]. RAD51 is a key enzyme, required to form RAD51-single-stranded DNA (ssDNA) filaments from DSBs, promoting strand invasion into a homologous duplex for the initiation of repair [2]. RAD51-associated protein 1 (RAD51AP1), an accessory to RAD51 [1,3], greatly enhances recombinase activity, stimulating a RAD51-mediated D-loop reaction [4].

Recently, our group published evidence for a novel role of RAD51AP1 in lung and ovarian cancers, with up-regulation of RAD51AP1 in patient tissue and blood samples compared to control samples at mRNA level [3]. Corroborative data, in silico analyses, demonstrated poorer overall survival (OS) in patients with high RAD51AP1 expression. In addition, suppression of RAD51AP1 by siRNA reduced cell proliferation of ovarian cancer (OvCa) cells in vitro [3]. Confirmatory data and emerging evidence for the oncogenic potential of RAD51AP1 in other cancers is strengthening the relevance of this molecule [1,5,6,7].

A recent study of a phase-I trial assessing the use of selenium plus chemotherapy in gynecological malignancies, such as ovarian, fallopian tube, and peritoneal cancers, revealed post-treatment down-regulation of RAD51AP1 in clinical samples; this was also shown using in vitro models [8]. Moreover, RAD51AP1 appears as one of an eight-candidate gene-signature that is of prognostic value to patients with lung adenocarcinoma (LUAD) [9].

In the present study, we measured the protein expression of RAD51AP1 in a tissue microarray of OvCa, and further expanded on our original observations regarding the role of RAD51AP1 by performing an enrichment analysis in an OvCa cell line where RAD51AP1 has been silenced. Changes in gene expression following RAD51AP1 silencing have been identified and differentially expressed genes (DEGs) matched to biological pathways.

## 2. Materials and Methods

### 2.1. Data Analysis

We leveraged information associated with gene expression data and patient phenotype from The Cancer Genome Atlas (TCGA) research network ((cancer.gov/tcga), accessed on 30 September 2021) and the Genotype Tissue Expression (GTEx) consortia project ((gtexportal.org), accessed on 30 September 2021) as recorded in the Xena repository, maintained by the University of California Santa Cruz [10]. A summary of the associated patient phenotype data is available in Table 1. Data was extracted from the TCGA-TARGET-GTEx pan-dataset cohort, that records normalized gene expression profiles from TCGA and GTEx in units of log_2_ (norm_count + 1) [11]. 

### 2.2. Immunohistochemistry (IHC)

IHC was performed on a paraffin-embedded tissue microarray slide (BC11115d, US Biomax Inc., Derwood, MD 20855, USA), containing 100 cores (90 OvCa and 10 normal adjacent ovarian tissue samples abbreviated to NAT; Supplementary Materials Table S1). Appropriate ethical approvals were in place for the use of these tissue samples, including using the Health Insurance Portability and Accountability Act (HIPAA) approved protocols. The protocol below is a brief summary, which is fully elaborated in Kerslake et al. [12]. Primarily, the slide underwent de-paraffinization and antigen retrieval. Blocking was performed in a humidity chamber with 5% BSA in PBS for 1 h at room temperature (RT). Primary antibody (ab101321, Abcam, UK) was added on the slide at 1:200 in 5% BSA in PBS in a humidity chamber and incubated overnight at 4 °C. Anti-rabbit secondary antibody from the Zytochem Plus HRP-DAB Kit (HRP008DAB-RB, Zytomed Systems, UK) was added to the slide and incubated for 1 h at RT in a humidity chamber. Streptavidin–HRP conjugate from the same kit was added to the slide and incubated for 50 min at RT in the humidity chamber. The DAB solution was prepared as per manufacturer’s instructions, dispensed onto the slides, and incubated for 10 min at RT. Counterstaining with Harris’ haematoxylin was followed before dehydration, and the slide sealed with a coverslip and DPX mounting medium (D/53330/05, Fisher Chemical, UK). Each core was scored from three individual researchers for the degree of RAD51AP1 staining on a scale of 0 to 4 (0: ≤10% stained; 1: 10–25% stained; 2: 25–30% stained; 3: 50–75% stained; and 4: ≥75% stained) using a light microscope at 10x magnification (Olympus BX51). 

### 2.3. siRNA for RAD51AP1

The SKOV3 cell line (ATCC, USA) was used as an in vitro model for OvCa. Cells were grown in Roswell Park Memorial Institute (RPMI) media supplemented with 10% FBS (Gibco) and 1% penicillin/streptomycin (Gibco). Cell lines were cultured at 37 °C, in 5% CO_2_ environment. siRNA targeted to RAD51AP1 (SMARTpool: ON-TARGETplus, Dharmacon, CO, USA) was used to suppress RAD51AP1 gene/protein expression in SKOV3 cells, as previously described [3].

### 2.4. Microarray and Gene Validation

Microarray of control and siRNA treated SKOV3 cells followed by RT-qPCR for validation of target genes took place, as previously described [3].

### 2.5. Gene Enrichment

FunRich v3.1.3 [13] was used for gene enrichment. FunRich analyses biological processes, cellular components, protein domains and molecular functions, expression sites, biological pathways, and transcription factors and provides a clinical synopsis of phenotypic terms using common genomic databases. 

### 2.6. Statistical Analysis

We used the Student’s *t*-Test statistical analysis as implemented in the R ggpubr library (v.0.4.0) to compare the average gene expression across various patient age and stage groups using data from the TCGA. One-way ANOVA followed by Tukey’s Multiple Comparison Test and the Student’s *t*-Test with significance determined at the level of *p* < 0.05 as implemented in GraphPad Prism were used to test the significance of the RAD51AP1 protein expression in ovarian cancer across various patient groups. We computed all against all gene expression, age and stage correlation and quantified it using the Pearson correlation coefficient as implemented in the R standard statistical library. 

Patient data from TCGA stratified by age and stage were used to compute variance within and between groups based on individual gene expression. Separation was computed as the ratio of between variance and within group variance, with the top scoring separation coefficient indicating the best gene expression pattern that can be used to differentiate between these patient phenotypes.

## 3. Results

### 3.1. RAD51AP1 Is Overexpressed in Ovarian Cancer

**Figure 1:**
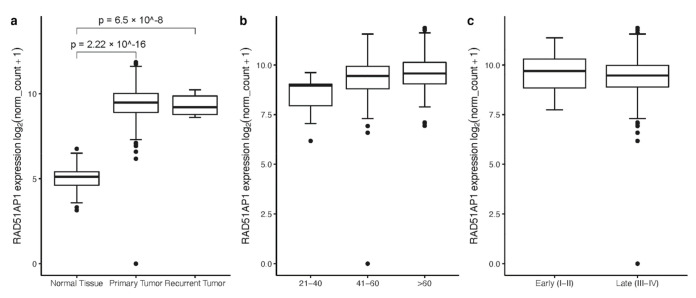
RAD51AP1 expression in (**a**) ovarian cancer, (**b**) as function of patient age at initial diagnosis (<40 years: n = 8, 41–60 years: n = 212, >60 years: n = 206) and (**c**) as function of stage (Early (I and II) stage: n = 27, Late (III and IV) stage: n = 399). RAD51AP1 is overexpressed in tumor (both primary (n = 419) and recurrent (n = 8)) compared to normal tissue (n = 88).

Leveraging the available expression data for OvCa from TCGA and GTEX projects, we investigated the differential expression pattern of RAD51AP1 in 419 primary (diagnostic) and eight recurrent OvCa samples as well as normal ovarian tissue (n = 88). In agreement with previous studies, RAD51AP1 is significantly up-regulated in primary and recurrent ovary tumors compared to control tissues (Figure 1a). Exploring the transcriptional landscape as functions of cancer stage and patient age at diagnosis (Figure 1b,c), we observed no significant difference with respect to staging (*t*-Test *p* = 0.18). Stratification based on the patient age, showed a small significant difference in the expression profiles for the under 60 vs. the over 60 years group (*t*-Tests: *p* = 0.034 for 41–60 vs. >60 and *p* = 0.049 for 21–40 vs. >60 years). 

We then performed a multivariate analysis to compare tumor stage, and age, with the levels of RAD51AP1 in OvCa (Supplementary Materials Figure S1).

### 3.2. RAD51AP1 Protein Is Aberrantly Expressed in Ovarian Cancer

**Figure 2:**
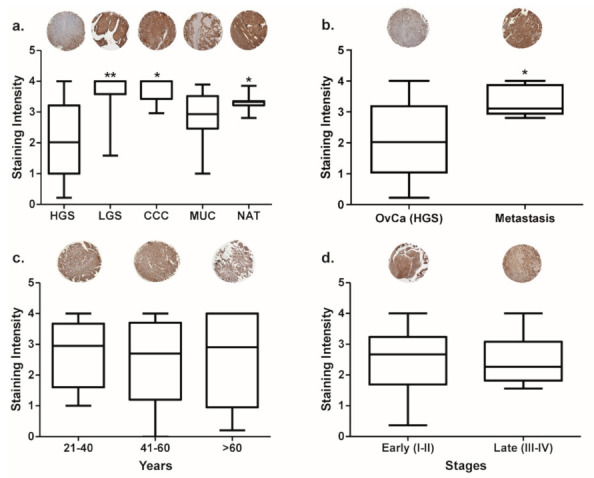
RAD51AP1 protein expression in ovarian cancer (OvCa) patients. A tissue microarray containing 90 unique OvCa patient samples and 10 normal adjacent tissues (NAT) was stained with RAD51AP1 antibody using immunohistochemistry (IHC). Staining intensity values measure the level of RAD51AP1 staining (brown): 0 ≤ 10% stained, 1 = 10–25% stained, 2 = 25–30% stained, 3 = 50–75% stained and 4 ≥ 75%. The cohorts were grouped based on: (**a**) pathological diagnosis: high grade serous (HGS), low grade serous (LGS), clear cell carcinoma (CCC), mucinous (MUC) OvCa and NAT, (**b**) lymph node (LN) metastasis compared to ovarian cancer (OvCa), (**c**) age, and (**d**) early (I and II) and late (III and IV) stages. The images of the cores that are presented above the plots are representative of the group and were taken at 10× magnification. Presented *p*-values were obtained using ANOVA and *t*-Test respectively; * *p* < 0.05, ** *p* < 0.001.

Τo determine the protein expression of RAD51AP1 in different ovarian tumors, IHC staining was performed on a tissue microarray slide containing 100 cores (90 OvCa patient samples and 10 NAT). These cores are from different patients of differing ages (22–69 years), stages (I–IV) and OvCa types. A range of histological subtypes of OvCa were represented: high grade serous (HGS), low grade serous (LGS), clear cell carcinoma (CCC), mucinous ovarian and metastatic OvCa retrieved from lymph nodes (LN). There was no significant difference in RAD51AP1 protein expression between different age groups or stages (Figure 2c and Figure 2d, respectively). However, RAD51AP1 expression was higher in LGS, CCC and NAT (Figure 2a) cores in comparison with HGS OvCa. Furthermore, RAD51AP1 expression was significantly higher in metastatic HGS OvCa of the lymph nodes compared to the primary site OvCa (Figure 2b). There was no significant difference of RAD51AP1 protein expression between different age groups or stages (Figure 2c and Figure 2d, respectively).

### 3.3. Differential Expression of Genes following Silencing of RAD51AP1 In Vitro

**Figure 3:**
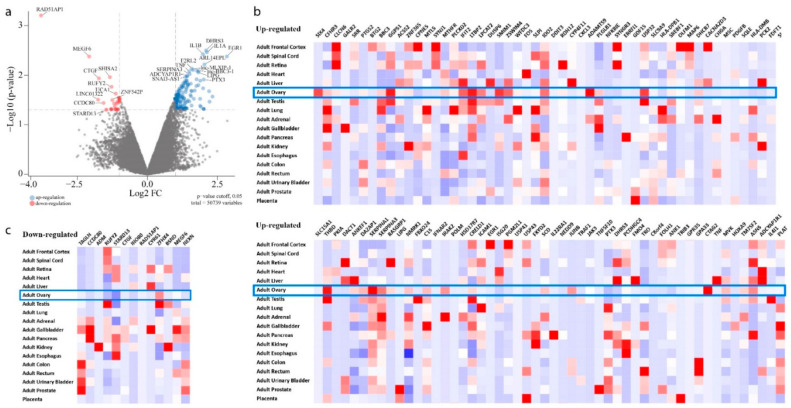
Differentially expressed genes (DEGs) in response to siRNA RAD51AP1 in SKOV3 OvCa cells. (**a**) Volcano plot, false discovery rate (FDR) threshold, FDR = 0.05. Horizontal/vertical lines, both log_2_FC = 1, represent thresholds for up/down-regulated DEGs. Genes with a −log_10_(p-value) > 2 are presented. Heatmaps displaying the (**b**) up-regulation and (**c**) down-regulation of these DEGs in different solid tissues; ovarian tissue highlighted in blue.

Following knockdown of RAD51AP1 in SKOV3 cells, 193 differentially expressed genes (DEGs) were identified (23 down-regulated and 123 up-regulated genes, Figure 3a). As expected, RAD51AP1 (red, top left, Figure 3a) has the highest down-regulation after silencing. To investigate the expression of these DEGs further, two heatmaps were produced using FunRich to display the up-regulated (Figure 3b) and down-regulated (Figure 3c) genes and their expression in solid tissues.

**Figure 4:**
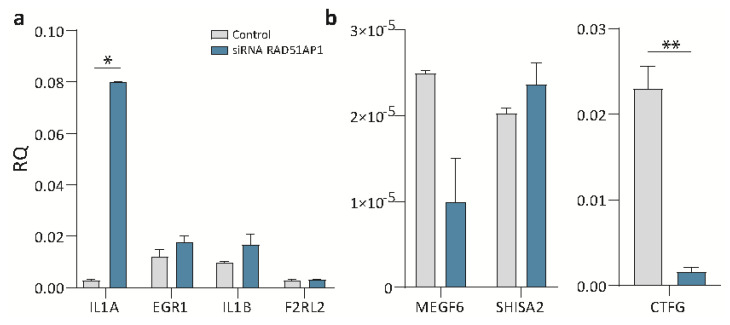
Validation of microarray data using RT-qPCR. (**a**) Up-regulation of expression in three of the four genes, with IL1A showing statistical significance (* *p* = 0.0175) and no change seen in F2RL2. (**b**) Down-regulation was noted in two out of three genes tested (MEGF6 and CTFG, but not SHISA2), with CTFG being significantly reduced in expression (** *p* = 0.0098). IL1A: interleukin 1 alpha, EGR1: early growth response 1, IL1B: interleukin 1 beta, F2RL2: coagulation factor II thrombin receptor like 2, MEGF: multiple EGF like domains 6, SHISA2: Shisa family member 2, CTGF: connective tissue growth factor.

In order to validate the outcome of the microarray analyses, certain up- and down-regulated genes were selected, and transcription levels were assessed using RT-qPCR. Validation of the genes identified from the microarray data generally show consensus, although two genes, namely F2R2L and SHISA2, showed little or no difference in expression in the siRNA treated cells (Figure 4).

### 3.4. Functional Analysis

**Figure 5:**
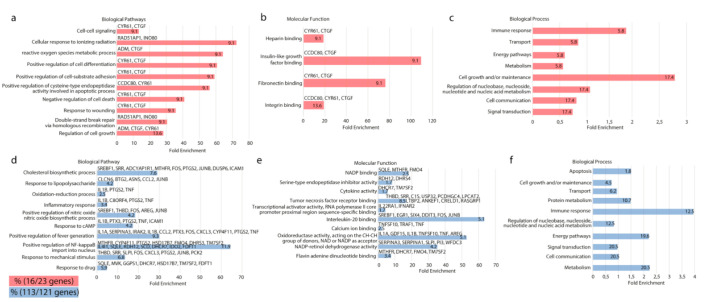
Differentially expressed genes (DEGs) identified from the silencing of RAD51AP1 were investigated for involvement in biological pathways (**a**,**d**), molecular function (**b**,**e**) and biological processes (**c**,**f**). The values on the bars indicate the percentage of genes associated with functional features, while the specific genes involved are labeled above. These 16 down-regulated genes (red) and 113 up-regulated genes (blue) were identified using FunRich. The percentage of genes identified involved in the associated pathways are indicated within the bar.

DEGs with cut-off criteria at *p* < 0.05 and [Log_2_FC] > 1 were selected for subsequent functional analysis. Accordingly, 16 of the down-regulated genes (Figure 5, red) and 113 of the up-regulated genes (Figure 5, blue) were located using the FunRich database. Subsequent pathway and process enrichment analysis was carried out using FunRich (funrich.org) and DAVID (david.ncifcrf.gov) with the following ontology sources: gene ontology (GO) biological pathways (Figure 5a,d), GO molecular functions (Figure 5b,e) and GO biological process (Figure 5c,f) [14].

**Figure 6:**
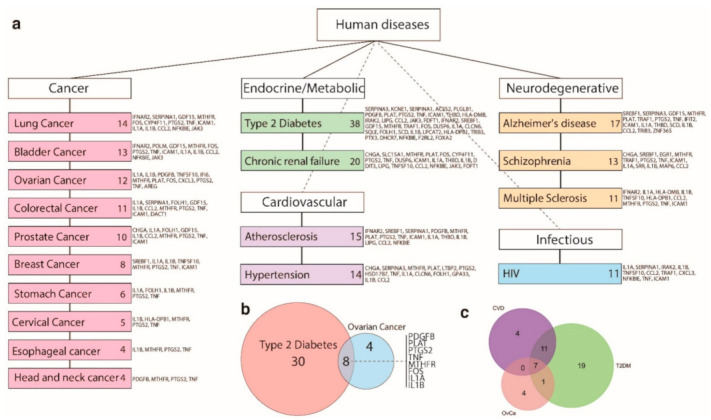
Using DAVID, up-regulated genes identified through RAD51AP1 silencing were functionally characterized through identification of their involvement in human diseases, including cancer, endocrine/metabolic-based disorders, and neurodegenerative diseases. (**a**) Through this analysis, it was identified that 96 (out of 122) of these genes were involved in human disease pathways, such as cancer and dysfunction in endocrine/metabolic pathways. The number of genes identified in each specific disease are alongside each pathway; (**b**) Venn diagram showing the overlap of genes in ovarian cancer (12 genes) and type 2 diabetes (38 genes), demonstrating eight common genes listed on the right side; (**c**) Venn diagram depicting a seven-gene signature overlap between ovarian cancer, type 2 diabetes and cardiovascular disease.

Using DAVID, the up-regulated DEGs were further investigated through identification of their involvement in human diseases (Figure 6). Of the 121 up-regulated DEGs, 96 were associated with endocrine, cardiovascular, neurodegenerative disorders, and cancer. Approximately 40% (38 out of 96) have reported associations with type 2 diabetes mellitus (T2DM), 29 out of 96 for cardiovascular disease. Twelve genes have reported associations with OvCa, seven of which were common to cardiovascular and T2DM. Genes for three inflammatory cytokines (IL1α, IL1β and TNFa) were among the seven up-regulated genes in both T2DM and OvCa [14]. Additionally, Fos, a well-known proto-oncogene was up-regulated in T2DM and OvCa but not cardiovascular disease [15]. We subsequently performed correlation analyses of the 12 candidate genes with RAD51AP1, where numerous genes exhibited weak albeit significant positive correlations (Supplementary Materials Figure S2). Furthermore, we have looked at stratification power of individual gene expression profiles and computed the variance within, between and separation coefficient for age groups (21–40, 41–60 and >60) and stages (I, II, III and IV) (Supplementary Materials Table S2). We observed that RAD51AP1 has the highest scoring separation coefficient for age and second highest for stages, indicating that its expression pattern is the best discriminant across these patient phenotypes.

**Figure 7:**
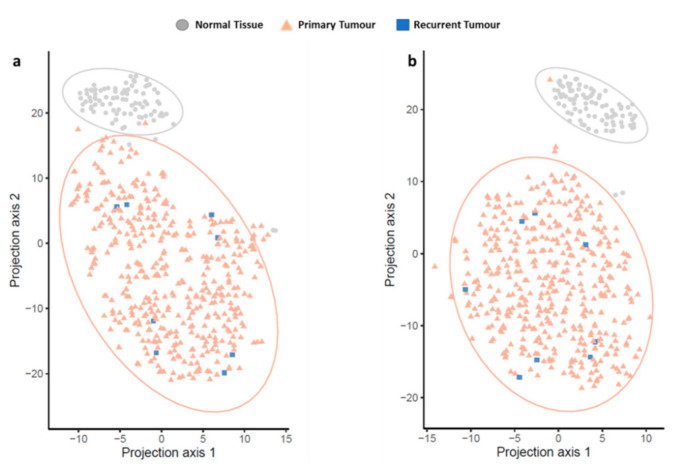
Tumor and normal tissue classification potential revealed by t-distributed stochastic neighbor embedding (t-SNE). Colored triangles and squares/rectangles represent ovarian tumor samples (n = 427), whereas grey circles represent normal ovarian tissue samples (n = 88). (**a**) Represents the expression matrix of the identified eight common genes in The Cancer Genome Atlas (TCGA) and the Genotype-Tissue Expression (GTEx) project embedded using t-SNE. (**b**) Represents 12 prognostic power gene expression matrix in TCGA and GTEx embedded using t-SNE.

Building on the differential expression analysis, we tested the OvCa diagnostic power for the eight genes associated with both OvCa and T2DM, as well as the 12 gene-set commonly found in OvCa. To this end, we used t-distributed stochastic neighbor embedding (t-SNE) dimensionality reduction method to discriminate between normal and tumor samples using the gene expression profiles (Figure 7). Overall, we identified a robust discrimination power for both sets of genes to identify patients with cancer vs. normal healthy individuals, with the larger set of 12 genes showing an improved differentiation between the two groups. 

## 4. Discussion

The present study complements our previous findings on the gene expression of RAD51AP1 in lung and ovarian cancers [3], and provides novel insight into the protein expression of RAD51AP1 in OvCa, as well as potential signaling changes due to silencing of the RAD51AP1 gene in vitro. Based on emerging studies, RAD51AP1 is implicated in multiple signaling pathways in health and disease. For example, we have shown potential involvement of mTOR signaling in OvCa, whereas Zhao et al. have shown a relationship between RAD51AP1 and in transforming growth factor β (TGF-β)/Smad signaling pathway. Using scatter plots, a significant positive correlation between RAD51AP1 with SMAD2-4 and TGFBR1 was observed [5]. This can be of increasing importance in OvCa, given that TGF-β can exert pro-tumorigenic effects, involving epithelial–mesenchymal transition (EMT) [16]. Using the latest data from TCGA and GTEX, we have demonstrated that RAD51AP1 is not only overexpressed in OvCa compared to controls, but also in metastatic sites compared to controls. Overexpression of RAD51AP1 has been described in other cancers, notably cholangiocarcinoma [17], hepatocellular carcinomas [7] and acute myeloid leukemia with complex karyotypic abnormalities [18]. Overexpression of RAD51AP1 in ovarian, breast and lung cancer has been generally associated with poorer overall survival [3,19]. In a study where two ovarian cancer cell lines were used (HEY and SKOV3), silencing of RAD51AP1 significantly inhibited the cell proliferation over 3 days [5] corroborating our initial findings in SKOV3 cells. Moreover, the migratory and invasive capacity of SKOV3 and Hey cells was compromised when they were transfected with siRAD51AP1, mirroring a wound healing assay where down-regulation of RAD51AP1 led to a decrease in the closure of the artificial gap [5].

Here we have demonstrated that there are no apparent differences in RAD51AP1 protein expression with respect to age or stage, in line with in silico gene expression data. RAD51AP1 expression was lower in HGS OvCa samples when compared to other histological subtypes (LGSC, clear cell as well as mucinous carcinoma) as well as the NAT. A limitation of this study is the absence of normal ovarian tissue for comparison given that NAT has been shown to bear unique characteristics, differentiating it from both healthy and tumor tissue [20].

Unexpectedly, there was a marked overexpression of RAD51AP1 in lymph node metastatic HGS OvCa tissue compared to primary HGS OvCa material. Clinical studies have shown that OvCa patients with isolated lymph node relapse (ILNR), have a significantly prolonged OS compared with patients with extra-nodal relapse and a reported median post-relapse survival of 2.5 to 4 years [21]. Overexpression of RAD51AP1 implies efficient homologous repair abilities, and a poorer prognosis. However, it is possible that the increase in RAD51AP1 found in the ILNR cohort is a secondary effect to a yet unknown factor prevalent in the lymphoid tissue environment. Molecular characterization of material from 49 cases of patients with ILNR failed to reveal any differences in the incidence of BRCA1/2 mutations or CCNE copy number gains when compared with OvCa tissue from matched patients with extra-nodal relapse sites [21].

Exploration of the DEGs identified by silencing RAD51AP1 in SKOV3 cells demonstrates many associations with endocrine and cardiovascular disorders. Prolonged inflammation is a well-described context for the emergence of many cancers and is associated with metabolic disorders, such as T2DM [22,23] and atherosclerosis [24]. Following RAD51AP1 silencing, of the eight up-regulated genes common to OvCa as well as these conditions, there are examples of key inflammatory cytokines, IL1α, IL1β and TNF in addition to the transcription factor, NFκB, known to regulate the aforementioned inflammatory cytokines [23,25]. Other mediators of inflammation are also up-regulated, including PTGS2, which codes for the COX-2 enzyme that promotes prostaglandin production and correlates with VEGF expression [26]. It is possible that these up-regulated pathways are adaptive survival mechanisms due to the silencing of RAD51AP1, and future studies are required to explore this further.

In line with the in vitro findings that silencing RAD51AP reduces OvCa proliferation, the commonly occurring down-regulated DEGs in the silenced SKOV-3 cells include cysteine-rich 61 (CYR6), connective tissue growth factor (CTGF), MEGF and UCA1. Cyr61 is associated with tumorigenesis and progression in gynecological carcinomas [27]; serum levels of Cyr61 are higher in advanced stage OvCa than in early stages [28]. Both MEGF and CTGF, an extracellular matrix (ECM) remodeling protein, have been shown to have roles in epithelial mesenchymal transition (EMT) and invasion, promoting cancer progression and metastasis [29,30]. UCA1 is another recognized oncogene, which has significantly higher expression in OvCa, and is associated with poorer OS; it is also involved in proliferation, migration and invasion as well as increasing resistance to paclitaxel [31,32].

There are a few limitations to be considered in interpreting these findings. More DEGs could have been validated at gene or protein level, including CTGF and CyR61, given their role in cell invasion and proliferation. Repeating the silencing experiments in other OvCa cell lines additional to SKOV-3 would improve understanding of integral RAD51AP1 related genes. In particular, exploring cell lines with BRCA1/2 mutations or other known HR deficiencies may improve our understanding of the role of RAD51AP1 in the context of HR in OvCa. Up/down-regulation of genes as a consequence of silencing RAD51AP1 is not necessarily proof of activity or inactivity of the transcribed proteins, but it is encouraging to see differential expression in molecular areas that fit with inflammation and cancer. 

To summarize, apart from RAD51AP1 being an accessory to RAD51, stimulating its activity and protecting against DNA damage [1], it should be noted that it has pleiotropic actions. Indeed, Pires et al., have proposed a model where RAD51AP1 can facilitate malignant/metastatic cells to overcome replication stress, by providing them with a growth advantage over normal cells [3]. Apart from its involvement in ovarian cancer, in our original study, we have also shown that RAD51AP1 is overexpressed in lung cancer, and silencing RAD51AP1 in vitro inhibits cell proliferation in a human lung cancer cell line [3]. Similarly, RAD51AP1 is up-regulated in intrahepatic cholangiocarcinoma (ICC) and knockdown of the gene resulted in suppression of cell proliferation [17]. RAD51AP1 is also significantly up-regulated in triple negative breast cancer and its overexpression is associated with poor outcome, suggestive of prognostic value in this malignancy [33]. More recently, RAD51AP1 was one of four other genes (*EXO1*, *TRIM59* and *SEPT3*) that can be an independent protective prognostic factor in patients with basal breast cancer [34]. Apart from its prognostic value, the siRNA data also points towards an interesting therapeutic target. For example, in colorectal cancer, it can act as a sensitizer to chemotherapy, and *Rad51ap1*-deficient mice were found to be protected against CRC [35].

## 5. Conclusions

The overlap of changes in gene expression after the silencing of RAD51AP1 with genes associated with T2DM and CVD, such as atherosclerosis, lends credence to the potentially common microenvironments found in these conditions. Indeed, there is a documented correlation between T2DM and an increased risk for gynecological cancers, including OvCa, for which there is recognition of shorter relapse-free intervals and reduced survival [36,37]. High glucocorticoid receptor expression has been shown in many solid tumors, including OvCa, and again correlates with reduced progression free survival [38,39]. However, the extent of this may be related to confounding variables, such as obesity or common biological pathways, such as IGF signaling, dysregulation of ovarian steroid hormones and increased insulin levels [36]. In the same way, utilizing therapeutic strategies that improve outcomes for patients with T2DM and atherosclerosis may also have benefits in OvCa. Recent studies have reported an association between metformin use in patients with concomitant T2DM and improved outcomes from ovarian cancer [36,37]. Another example involves the use of relacorilant, an antagonist of the glucocorticoid receptor, which has been shown to improve outcomes when given with paclitaxel to patients with platinum resistant/refractory ovarian cancer [40]. Furthermore, it will be intriguing to ascertain if the eight common differential gene signatures could be of predictive value. 

## Figures and Tables

**Table 1:** Summary of clinical phenotypes for patient samples from TCGA and GTEx.

Feature	TCGA	GTEx
Total Samples	427	88
Normal tissue	-	88 (100%)
Primary tumor	419 (98.13%)	-
Recurrent tumor	8 (1.87%)	-
Clinical stage		
Stage I	1 (0.23%)	-
Stage II	26 (6.09%)	-
Stage III	336 (78.69%)	-
Stage IV	63 (14.75%)	-
Age range (years)	30–87	-
<40	8 (1.87%)	-
41–60	212 (49.65%)	-
>60	206 (48.24%)	-

## Data Availability

RNAseq data can be provided upon reasonable request.

## Supplementary Materials

The following supporting information can be downloaded at: https://www.mdpi.com/article/10.3390/jpm12020201/s1, Table S1: List of ovarian cores used for tissue microarray, Table S2: Variance between and within age and stage patient groups of the 12 candidate genes for OvCa, Figure S1: Multivariate analysis of RAD51AP1 gene expression as function of age and clinical stage. Pearson correlation coefficient was used to evaluate the correlation between patient age and the gene expression, Figure S2: Cross-correlation of gene expression for 12 candidate genes. Pearson correlation coefficient was used to evaluate the co-expression between any two genes.
