# Supplementary figures and images for: Differential Expression of RAD51AP1 in Ovarian Cancer: Effects of siRNA In Vitro
Source: J Pers Med. 2022 Feb 1;12(2):201. doi: 10.3390/jpm12020201 (PMC8876735; doi:10.3390/jpm12020201)

Supplementary Figure S1

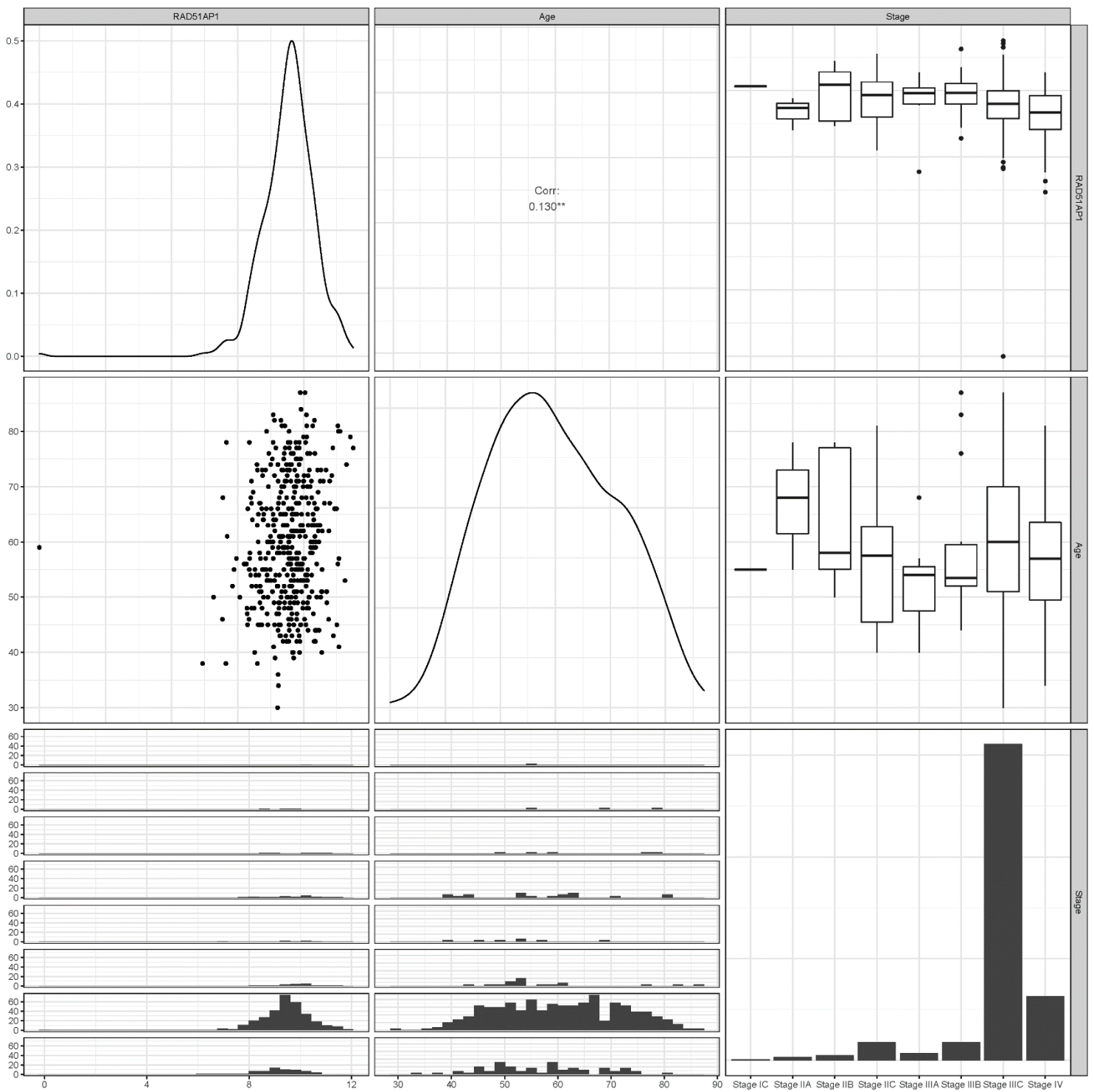

Supplement: Supplementary file 1 [file jpm-12-00201-s001.zip › Supplementary Figure S1.pdf]

### Supplementary Figure S2

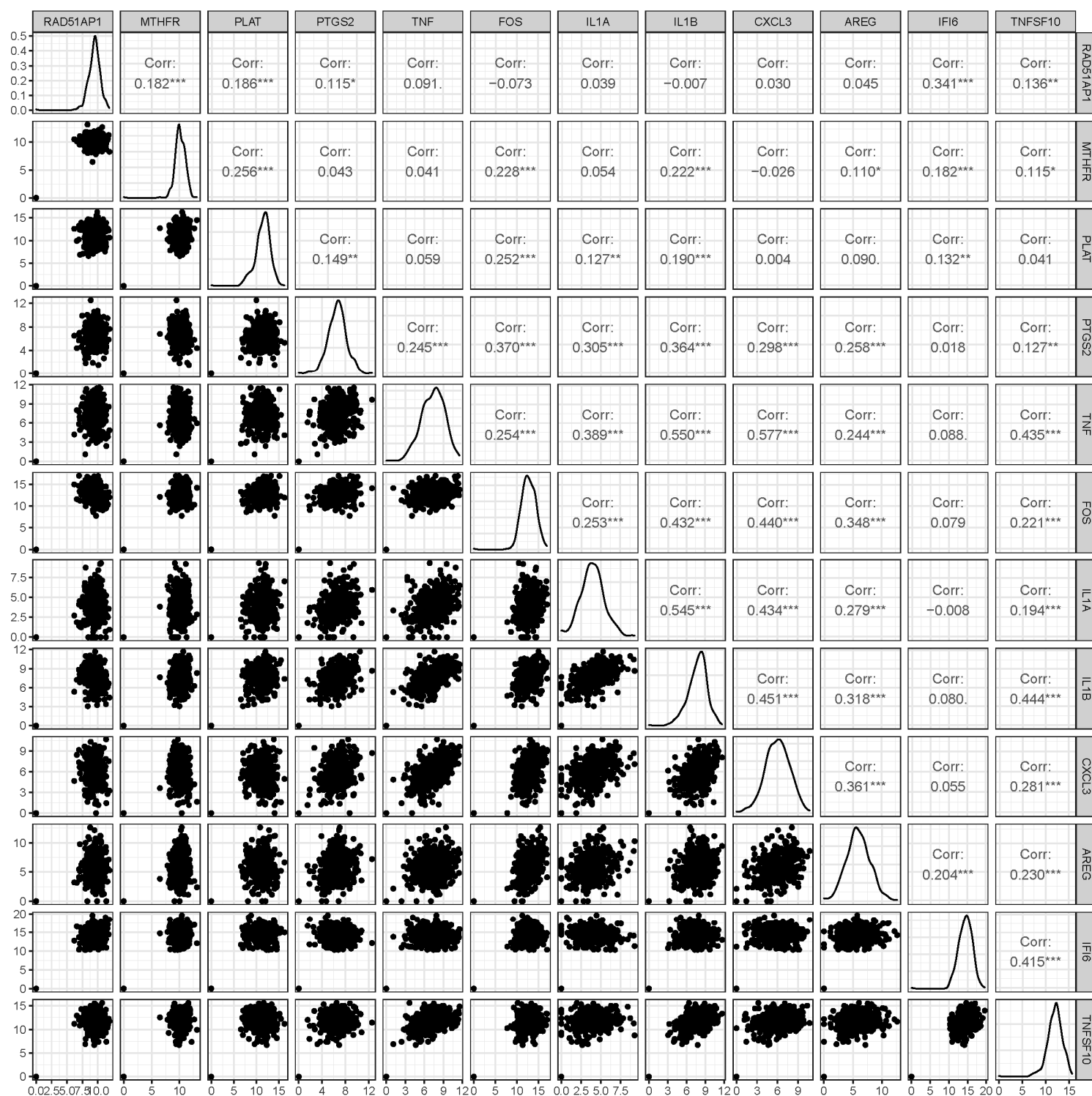

Supplement: Supplementary file 1 [file jpm-12-00201-s001.zip › Supplementary Figure S2.pdf]
